# Geologically-inspired strong bulk ceramics made with water at room temperature

**DOI:** 10.1038/ncomms14655

**Published:** 2017-03-06

**Authors:** Florian Bouville, André R. Studart

**Affiliations:** 1 Complex Materials, Department of Materials, ETH Zürich, 8093 Zürich, Switzerland

## Abstract

Dense ceramic materials can form in nature under mild temperatures in water. By contrast, man-made ceramics often require sintering temperatures in excess of 1,400 °C for densification. Chemical strategies inspired by biomineralization processes have been demonstrated but remain limited to the fabrication of thin films and particles. Besides biomineralization, the formation of dense ceramic-like materials such as limestone also occurs in nature through large-scale geological processes. Inspired by the geological compaction of mineral sediments in nature, we report a room-temperature method to produce dense and strong ceramics within timescales comparable to those of conventional manufacturing processes. Using nanoscale powders and high compaction pressures, we show that such cold sintering process can be realized with water at room temperature to result in centimetre-sized bulk parts with specific strength that is comparable to, and occasionally even higher than, that of traditional structural materials like concrete.

## Abstract (web summary)

Milder conditions for processing ceramics is of interest for a range of uses. Here authors report room temperature sintering of nanoparticulate powders using a solution-assisted route, yielding parts with centimetre-sized bulk parts with specific strength comparable to and possibly surpassing that of traditional structural materials like concrete.

Densification of synthetic ceramic materials at mild conditions has been long pursued as a path to enable simultaneous processing of inorganic and organic constituents into structures unattainable through state-of-the-art approaches. Advances in this direction would also remarkably reduce the consumption of energy during fabrication and the cost of ceramic and hybrid parts. Attempts to replicate in synthetic systems some aspects of the biomineralization strategies that permit such densification process to occur in nature have led to the successful creation of thin inorganic films^1^,^2^,^3^ and particles^4^,^5^ at temperatures far below what is typically needed to densify ceramics by traditional technologies (>1,400 °C). Applying such mild processing approach to also produce bulk parts could revolutionize the field of hybrid materials by opening the possibility to explore novel strategies to economically co-process organic and inorganic building blocks into the intricate hierarchical architectures found in functional biological materials^6^. In contrast to the cell-mediated biochemical pathways underlying the biomineralization of living tissues, the geological formation of dense and strong carbonaceous rocks from inorganic particle sediments provide another natural route for the densification of ceramic-like materials at relatively mild temperatures and pressures^7^. An attractive feature of geological processes is that they do not require the complex chemistry involved in biomineralization and they involve driving parameters, such as composition, pressure and temperature, that are more readily applicable at large scales using state-of-the-art technologies. The challenge of implementing such natural processes in an artificial context is to conceive a method that reduces by orders of magnitude the geological timescales required for the densification of carbonate sediments.

Here we show that the densification of carbonate compacts can be drastically accelerated by decreasing the size of constituent particles to the nanoscale and utilizing higher pressures during the compaction process in the presence of water. Surprisingly, we discovered that carbonate materials densified at ambient temperature (∼25 °C) within timescales of less than an hour can be as strong as existing building materials and comparable in strength and stiffness to other relevant man-made structural and biological materials. Since synthetic bioceramics are often slightly soluble in water, we expect our cold sintering process to be particularly attractive for this class of ceramics and their composites, for which finite solubility in aqueous medium is also a beneficial feature for tissue regeneration and bio-integration. In addition to enabling the fabrication of bulk hybrid and ceramic parts at milder conditions, this technology applied to carbonates can potentially also have a double positive effect in future carbon-neutral societies, since it utilizes a potential CO_2_-capturing chemical resource^8^ to replace key construction materials of currently large carbon footprints.

Recent work on cold sintering processes applied to ceramics^9^,^10^ has demonstrated impressive densification of various inorganic materials at mild temperatures under hydrothermal conditions. Typically, temperatures in the range 120–180 °C have been utilized to promote densification of oxides through cold sintering. Although sintering of NaCl at room temperature has also been reported^11^,^12^, this salt is prone to densification at such mild conditions due to its very high solubility in water (360 g l^−1^). Instead, we present for the first time the accelerated sintering in water at 25 °C of an inorganic compound that is only moderately soluble in water (1.9 g l^−1^; ref. 13). This pushes further down the lower sintering temperature limit and eliminates the need for hydrothermal conditions to accomplish sintering of sparsely soluble compounds in water.

## Results

### Sintering mechanisms at room temperature

**Figure 1:**
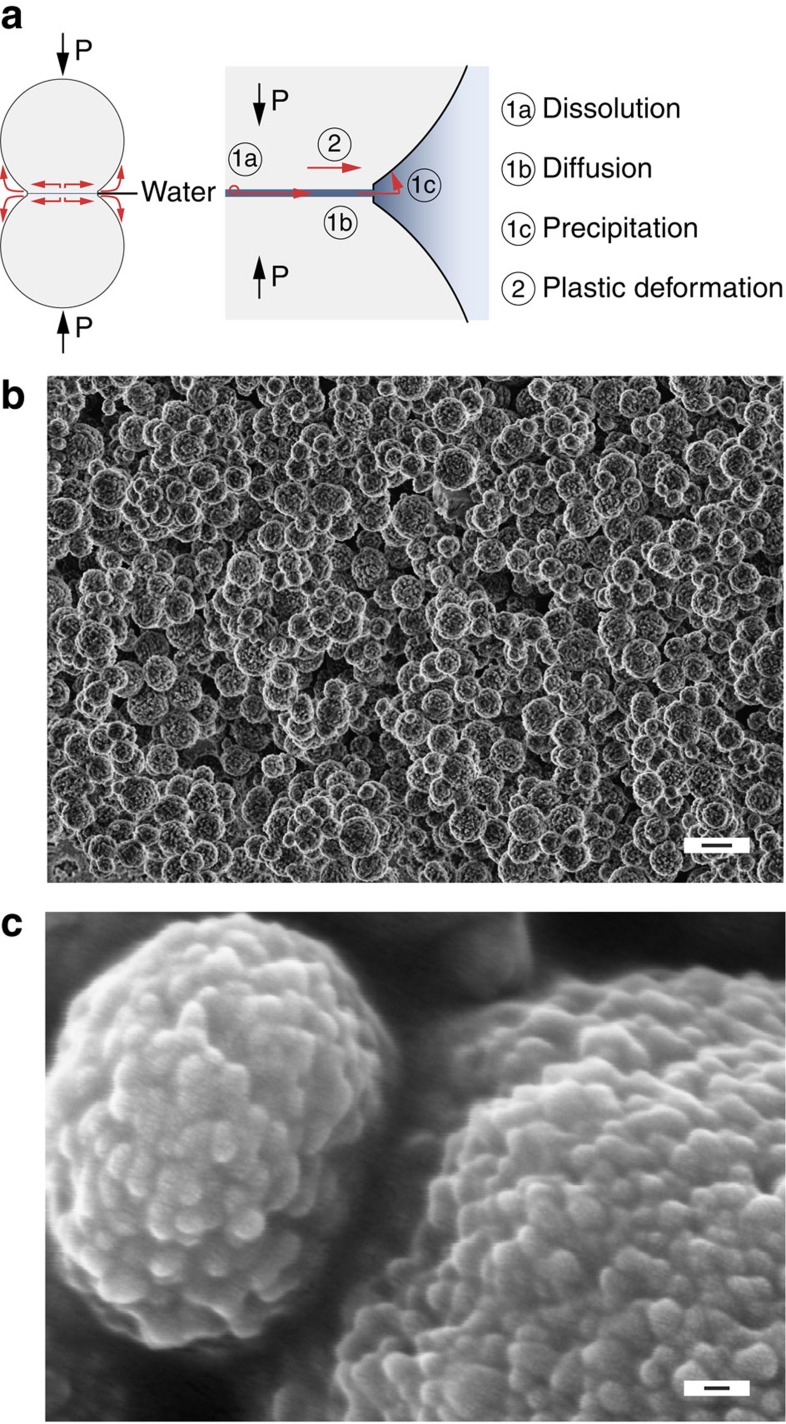
Vaterite particle morphology and possible transport mechanisms for cold sintering. (**a**) Schematics of the mass transport mechanisms around the contact point between particles subjected to an external mechanical load, *P*. (**b**,**c**) Scanning electron micrographs showing the hierarchical morphology of precipitated nanovaterite particles at different magnifications. Scale bar, 2 μm (**b**); 50 nm (**c**).

The basic physico-chemical mechanisms underlying the geological densification process of carbonate rocks in nature, known as cold sintering, may involve the different mass transport pathways schematically depicted in Fig. 1a (refs 12, 14, 15, 16, 17, 18, 19, 20, 21). The dissolution–precipitation process, referred to pressure solution creep in geology, relies on the transport of matter from the contact between touching particles to the surrounding liquid phase and eventually to nearby non-contacting surfaces. Such transport locally decreases the distance between the particle centres, enabling global shrinkage and densification. The motion of ions during pressure solution creep is driven by the high-stress concentration at the contact point (grain boundary) when the particle compact is subjected to an externally applied mechanical load. In response to such stress concentration, ions at the contact point dissolve from the solid particle into the interfacial water film, and eventually diffuse along the grain boundary before reaching the continuous liquid phase and precipitating on a non-stressed particle surface^12^. The dissolution at the particle contact point is favoured by the high local compressive stress at this region as compared to other locations of the particle, alike the dissolution–precipitation processes expected for liquid phase sintering of ceramics at much higher temperatures, typically above 1,200 °C (ref. 22). In addition to the dissolution–precipitation mechanism, plastic deformation through dislocation motion and/or viscous flow of the solid phase might also contribute to the densification process at sufficiently high applied pressures (Fig. 1a).

Well-established models that describe such densification mechanisms predict an increase in densification rate by reducing the particle size and increasing the externally applied stresses (see Supplementary Information)^14^,^16^,^23^,^24^. While the use of nanoparticles to accelerate the densification process of ceramic materials has been utilized for several decades^22^,^25^ and have recently also been exploited for pressure-assisted sintering at milder temperatures down to 150–200 °C (refs 26, 27, 28) or for binding at room temperature^29^, no evidence was shown thus far that this effect can also be harnessed to densify a sparsely soluble ceramic material into strong bulk compacts with water at ambient temperature.

To investigate the cold sintering of nanoscale carbonate agglomerates at room temperature, we synthesized vaterite nanoparticles following a protocol originally proposed by Parakhonskiy *et al*.^30^ In this synthetic procedure, sodium carbonate and calcium chloride are used as sources of CO_3_^2−^ and Ca^2+^ ions, respectively. Vaterite nanoparticles are easily formed through simple mixing of these reactants in an aqueous solution of ethylene glycol. The resulting carbonate powder exhibits a unique hierarchical structure comprising 37 nm particles that are densely arranged into 0.6 μm spherical agglomerates (Fig. 1b,c and Supplementary Fig. 1). The size of the nanoparticles was confirmed by transmission electron microscopy observation (Supplementary Fig. 2). Although no secondary phase could be detected at the nanoparticle grain boundaries, this should be confirmed in future studies by more detailed structural analysis. The powder contains also a small fraction of micrometric cubic-shaped calcite crystals. After synthesis, the nanovaterite powder is washed with excess ethanol to remove the ethylene glycol before proceeding with compaction measurements.

### Room temperature compaction in water

**Figure 2:**
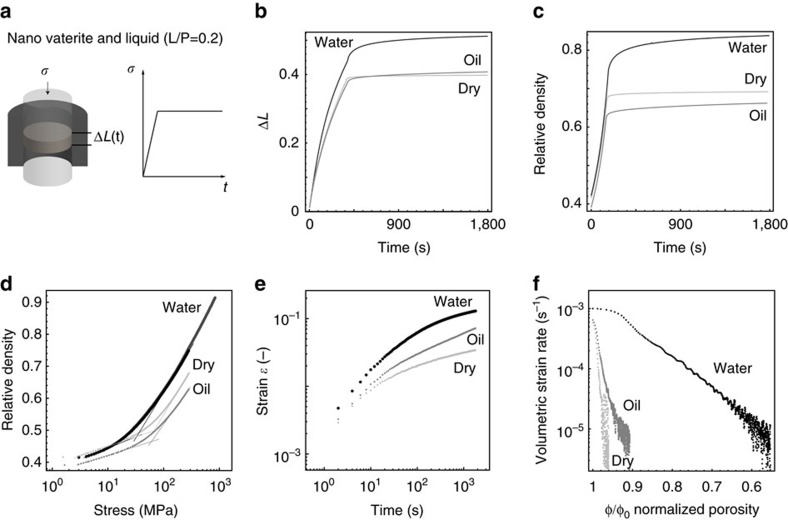
Uniaxial compaction of nanovaterite powder in different continuous liquid phases. (**a**) Schematics of the compaction set-up and the stress ramp applied during the experiments. *σ* is the externally applied stress, Δ*L* is the measured net dimensional change and *t* is the elapsed time. (**b**) Dimensional change as a function of time for powder compacts in water, paraffin oil or in the dry state subjected to a maximum applied stress of 280 MPa. (**c**) Densification behaviour and creep response of powder compacts in the presence of different continuous phases under an applied stress of 280 MPa. The theoretical density of vaterite was taken as 2.51 g cm^−3^. (**d**) Relative density as a function of applied stress for vaterite powders in the presence of three different media. The liquid-to-powder weight ratio was L/P=0.2. The measured yield stresses^37^ are 31 MPa, 70 MPa and 60 MPa for the compositions with water, dry and with paraffin oil, respectively. The two different curves presented for the composition with water were obtained by applying a maximum stress of 280 and 800 MPa. (**e**) Strain as a function of time for vaterite powders subjected to a constant pressure of 280 MPa in the presence of three different media. (**f**) Volumetric strain rate as a function of the normalized porosity. Φ_0_ refers to the porosity achieved at the beginning of the stress plateau for each composition.

Compaction tests were performed by applying an uniaxial mechanical load onto a vaterite-liquid mixture added to a cylindrical mould at an initial solid–liquid weight ratio of 0.2 (Fig. 2a). The mechanical load applied to the mixture was first increased at a displacement rate of 0.5 mm min^−1^ until the target maximum stress was reached. The dimensional change (Δ*L*) and respective uniaxial deformation (*ɛ*) along the loading axis were measured as a function of time during mechanical loading and while keeping the specimen under the constant target stress, *σ*.

Considering that an aqueous continuous phase is necessary to enable the dissolution of ions during pressure solution creep (Fig. 1a), we first conducted experiments in the presence or absence of water to elucidate the role of cold sintering in the compaction process. For that purpose, vaterite powders mixed with water, paraffin oil or in the dry state were compacted uniaxially at the same target stress of 280 MPa. To quantify the level of densification achieved in the compaction process, the obtained raw deformation data (Fig. 2b) were converted into relative density values, leading to the densification curves shown in Fig. 2c. Compacts containing water reach a striking relative density of 84 % at room temperature in a time frame of only 30 min. By contrast, the presence of air or paraffin oil as continuous phase increases the relative density to only 68% and 64%, respectively. As ion dissolution and liquid-induced plasticity should not occur in the oil or in air (dry), this confirms that dissolution–precipitation and/or plastic deformation processes activated by water are key mechanism in the densification process of the compacts.

The effect of water in promoting plastic deformation during mechanical loading can be quantified by analysing the compaction data in terms of density versus applied stress for the three compositions (see Fig. 2d). The plasticizing effect is evidenced by an approximately 50% lower-yield stress obtained when water is present compared to the dry powder and with paraffin oil. Water-induced plasticity is also known to affect the yield pressure of ceramic granules during compaction^31^. In contrast to the 50–55% relative density observed during compaction of clay-free granules, the densification of the vaterite nanopowder proceeds at a high rate up to relative densities as high as 90%. This surprising effect confirms the unusual behaviour of the water-assisted compaction of the nanopowder studied here.

To evaluate the effect of pressure solution creep on the densification behaviour of the vaterite powder, we also compare the strain of the compacts as a function of time at a constant pressure for compositions containing different media (Fig. 2e). Similar to the effect of pressure alone (Fig. 2d), water is also found to promote densification of the compact if the pressure is kept constant. The initial effect of water in the compaction process is thus further amplified if the samples are allowed to continuously deform through water-induced creep mechanisms. The creep of vaterite compacts at constant pressure can also be described in terms of the strain rate as a function of normalized porosity (Fig. 2f), as is typically used to report geological experiments. The much higher strain rates achieved in water provides clear evidence of the major role of cold sintering mechanisms (Fig. 1a) in enabling strong densification during compaction.

### Microstructural evolution

**Figure 3:**
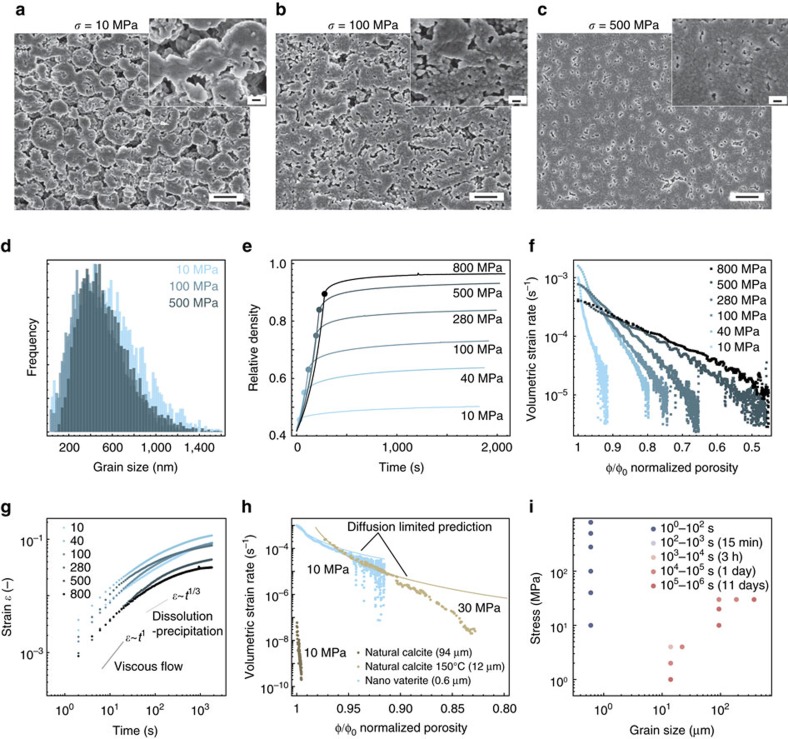
Densification behaviour of nanovaterite compacts and comparison with model geological calcite. (**a**–**c**) Scanning electron micrographs of vaterite compacts subjected to different external stresses. Scale bar, 1 μm (**a**–**c**), inset: 200 nm. (**d**) Grain size distribution of compacts obtained at 10, 100 and 500 MPa. The reported data refer to the size of the vaterite agglomerates. (**e**,**f**) Densification behaviour and creep response of nanovaterite samples measured at different applied stresses at weight ratio of liquid to powder of L/P=0.2. The filled circles in **e** mark the start of the stress plateau for each curve. (**g**) Logarithmic plot of the strain as a function of time during the constant pressure step at different stress levels. Different slope guides are plotted along with the data. The exponent *n* describing the time dependence of the strain seems to lie in between 1 and 1/3 within the first 60 s. The strain rates eventually slow down for longer elapsed times. (**h**) Comparison between the creep response of nanovaterite and model geological calcite. (**i**) Timescales required to increase the relative density of powder compacts by 0.4% as a function of the grain size and applied stress at room temperature.

The evolution of the nano- and microstructure of the powder at different compaction stages was examined using scanning electron micrographs obtained from samples subjected to distinct applied stresses (Fig. 3a–c). At the low applied stress of 10 MPa, the spherical morphology of the nanovaterite agglomerates is still recognizable and the large inter-agglomerate interstices constitute a major fraction of the overall porosity. A higher applied stress of 100 MPa leads to significant densification and deformation of the agglomerates. This effect is further enhanced at 500 MPa, where agglomerates are no longer visible and only residual porosity remains. Neck formation during the cold sintering process is expected to take place both between the nanoparticles and between the agglomerates. This is supported by the fact that the powder compacts achieve a relative density level (up to 90%) that is much higher than the random close packing density of approximately 64% that would be expected from the densification of only nanoparticles or agglomerates. Interestingly, the densification processes achieved by increasing the applied pressure is not accompanied by the grain coarsening typically observed during sintering of ceramics at high temperatures (Fig. 3d)^22^. This is an important advantageous feature of the cold sintering process, since large grain sizes have a deleterious effect on the mechanical strength of brittle materials^32^. We also observed that the metastable vaterite phase is preserved after pressing at 500 MPa and does not present a preferential crystallographic orientation, even at high pressures (Supplementary Fig. 1). Importantly, preliminary aging experiments of compacts in water revealed no phase transformation from vaterite to calcite within a time period of 2 weeks after compaction. Instead, 2–4 days are enough to transform such powder when immersed in water under stirring^30^. This suggests that the constrained environment within the dense compact prevents conversion of the carbonate into its thermodynamically stable phase.

### Effect of particle size and applied pressure

The dependence of the relative density on the applied stress at room temperature was further evaluated by performing compaction tests over a wide range of increasing pressures followed by a stress plateau (Fig. 3e). A remarkable relative density of 87% is achieved at room temperature at a pressure of 500 MPa. Such a pressure level can be easily applied to compact parts at the tens of centimetre scale using a standard industrial hydraulic press. At the highest applied pressure of 800 MPa, the final compact does not present a higher relative density compared to a sample pressed at 500 MPa. This suggests that the additional deformation visible in Fig. 3e for the specimen pressed at 800 MPa is mostly elastic and thus reversible. As observed for the experiments in different media (Fig. 2c,d and Supplementary Fig. 3a,b), the relative density of the powder compact was found to exhibit a logarithmic dependence on the applied stress (Supplementary Fig. 7), which qualitatively agrees with the behaviour expected for geological and synthetic materials^33^.

Further analysis of the compaction data reveal that the strain of the compacts (*ɛ*) is found to follow for all investigated pressures a power relation with respect to the elapsed time (*t*) of the form: *ɛ*=*t*^*n*^, where the exponent *n* varies between 1 and 1/3 within the first 60 s, followed by a slow down of the densification process at longer times (Fig. 3g). In classical liquid-assisted sintering models^22^,^34^, an exponent of 1 is expected when mass transport is dominated by viscous flow-induced particle rearrangement at an early densification stage. This is typically followed by a dissolution–precipitation process at the contact point between grains, which results in an exponent of 1/3 for the strain-time relation. While further studies are need to elucidate the dominant mass transport processes operating at different timescales and pressures, the observation of similar exponents in our time-dependent strain data suggest that liquid-assisted densification mechanisms that resemble classical sintering processes seem to govern the sintering of the vaterite compounds investigated in this work.

When the deformation response during the stress plateau is displayed in the form of creep curves (Fig. 3f), we observe that the strain rate of the compacts decreases from an initial value around 10^−3^ s^−1^ to a level in the order of 10^−5^ s^−1^ as densification proceeds. At any given relative porosity, higher strain rates are observed in compacts subjected to higher stresses, which explains the stronger densification of samples at elevated pressures. A comparison of our creep data with values reported in geological studies at a low applied stress of 10 MPa reveals that the nanovaterite compact (agglomerate and nanoparticle sizes of 0.6 μm and 37 nm, respectively) deforms at strain rates many orders of magnitude higher than those obtained for a coarser natural calcite sample (grain size of 94 μm, Fig. 3h)^23^. Since other important parameters like diffusion coefficient or solubility are the same or only change slightly among CaCO_3_ polymorphs^13^, this difference must arise from the more than 100 (1,000) times smaller sizes of the vaterite agglomerates (nanoparticles). Strain rates comparable to those measured for the nanovaterite at 10 MPa can only be achieved with geological model calcite by increasing the compaction temperature and pressure to 150 °C and 30 MPa, respectively, and by reducing the grain size to 12 μm (Fig. 3h)^15^.

Although multiple mass transport mechanisms might be involved in the cold sintering process (Figs 1a and 3g), the creep response of calcite and nanovaterite compacts subjected to stresses of 30 MPa and 10 MPa, respectively, were found to be reasonably described by a established pressure solution creep model (see Supplementary Information). For this estimation, dissolution–precipitation was assumed to occur predominantly between the vaterite agglomerates, as suggested by the necks formed between them in the SEM images shown in Fig. 3a (inset). By contrast, attempts to fit the creep data of nanovaterite specimens pressed at 40 MPa and above led to an overestimation of the strain rate by several orders of magnitude (see Supplementary Fig. 6). Such overestimation could be caused by an approximation in the model, which makes it only valid for relatively small local stresses at the contact point between particles^14^. Alternatively, this disagreement between model and experimental data might reflect the existence of other mass transport mechanisms that become more significant at high applied stresses. Studies on natural minerals^12^,^35^ usually indicate a coupling of dissolution–precipitation (pressure solution creep) mechanisms and plastic deformation at applied stresses higher than 100 MPa. This pressure threshold depends on the composition, grain size and temperature.

To gain insights into the effect of the particle solubility on the cold sintering process, we also performed compaction experiments using different aqueous media. Modification of the liquid medium to decrease carbonate solubility by using ultra pure water or increase particle dissolution by reducing the pH to 6 does not change the densification kinetics at high stresses (see Supplementary Fig. 8). This suggests that indeed a different limiting factor is at play at high pressures and sample densities. While further studies are needed to elucidate and quantify the dominant mechanisms at work at different applied pressures, our results clearly indicate that these mass transport mechanisms are activated and amplified when our nanoparticles are exposed to water (Fig. 2c). This contrasts with the response of natural materials with larger grains, for which plasticity and fracture are also present without water^12^.

Although the strain rates measured here are lower than estimates from simplified analytical models, the densification of nanovaterite compacts occurs within remarkably shorter timescales if compared to model geological specimens with larger grain sizes. Experimental timescales obtained for our vaterite specimens and literature values for calcite samples are shown in Fig. 3i for a wide range of grain sizes and applied stresses. Each dot in this plot corresponds to an experiment at a given grain size and pressure applied, whereas the dot filling colour is representative of the time this sample took to be compacted by an arbitrary value of 0.4% at a constant applied stress. The time values are thus indicative measures for the very early stage of the cold sintering process. Strikingly, reducing the grain size and increasing the applied stresses decreases the densification timescales from about 10 days to only a few seconds.

The compaction stresses and particle sizes covered in this study exploit a completely new parameter space compared to earlier investigations on geological model systems (Fig. 3i). This opens the opportunity to produce bulk ceramic parts with water at room temperature with relative densities comparable to those of state-of-the-art building and structural materials. The densification process takes place within economically viable timescales and is applicable to cheap and abundant raw materials that can even be used as CO_2_ sinks. The fact that it proceeds with water and at room temperature is a major advantage, which contrasts to the energy-intensive heat treatments beyond 1,400 °C typically employed in the ceramic industry.

### Strong and stiff cold-sintered carbonates

**Figure 4:**
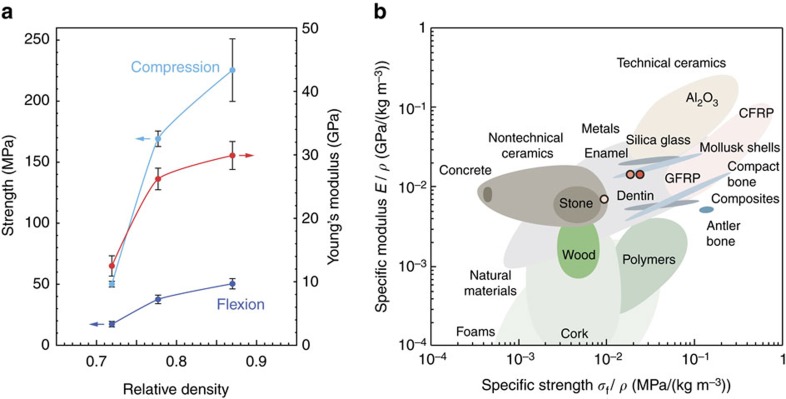
Mechanical properties of nanovaterite compacts. (**a**) Strength and elastic modulus of nanovaterite specimens as a function of the compact relative density. (**b**) Ashby diagram displaying the specific modulus and specific compressive strength of the nanovaterite compacts in comparison to other man-made and biomineralized materials^38^. Data for the nanovaterite materials are indicated by red, orange and pink circles, which correspond to specimens with 87%, 78% and 72% relative density, respectively.

The high relative densities achieved through high-pressure cold sintering of nanopowders translate into surprisingly high mechanical properties for compacts fabricated at room temperature without the addition of binders (Fig. 4a). At a relative density of 87%, the cold-sintered compacts exhibit an elastic modulus of 30 GPa combined with flexural and compressive strength of 50 MPa and 225 MPa, respectively. If compared with other materials classes (Fig. 4b), the dense nanovaterite compacts outperform many state-of-the-art construction materials like stone, concrete and wood, reaching higher-specific strength at comparable or even higher elastic moduli. This statement is valid even if size effects are taken into consideration (see relevant section under Supplementary Methods). It is important to note that this comparison is meant to provide an order of magnitude analysis, since the properties of concrete reported in the Ashby diagram (Fig. 4b) represent standard materials measured in the form of large samples and does not include high-performance formulations.

Remarkably, the cold-sintered bulk CaCO_3_ parts also reach strength and elastic modulus levels within the same range of those found for highly biomineralized natural materials like mollusk shells and enamel. Although this study is focused on calcium carbonate, we expect other nanoscale powders to also densify at room temperature under the range of pressures examined here. In addition to the nanoscale particles, a key additional requirement for the densification process to occur is to tune the chemistry of the aqueous medium to ensure a minimum solubility of the powder in the continuous liquid phase.

In conclusion, cold sintering of a nanoscale powder at high pressures enables the fabrication of strong and dense structural materials with water and at room temperature within timescales comparable to those of typical manufacturing processes. This simple up-scalable process offers an alternative pathway for the processing of inorganic materials under energy-inexpensive mild conditions and may allow fabrication of complex organic–inorganic architectures that mimic the design principles of functional biological materials.

## Methods

### Synthesis of nanovaterite agglomerates

The method used here to synthesize nanovaterite agglomerates was originally developed by Parakhonskiy *et al*.^30^ Briefly, two solutions comprising 20 vol% of distilled water and 80 vol% of ethylene glycol (>99%, Sigma-Aldrich) were first prepared. CaCl_2_·2H_2_O (EMSURE ACS, Reag. Ph Eur, Merck Millipore) was added to one solution, whereas NaCO_3_·10H_2_O (puriss. 99.5%, Sigma-Aldrich) was dissolved in the other. This resulted in water/ethylene glycol solutions containing 0.33 mol l^−1^ of either CaCl_2_ or NaCO_3_. A typical calcium chloride solution contained 8.00 g of water, 36.06 g of ethylene glycol and 4.66 g of CaCl_2_·2H_2_O. For the sodium carbonate solution, 8.00 g of water, 36.06 g of ethylene glycol and 9.06 g of NaCO_3_·10H_2_O were used. The two solutions were mixed quickly and kept under agitation at ∼600 r.p.m. using a magnetic stirrer for 2 h. The precipitated powder was washed two times with ethanol (94% denaturated with Toluene, Alcosuisse) through sequential centrifugation steps and eventually stored in ethanol if not used. All chemicals were used as received without further purification.

### Structural characterization

Cross-sections of the sample were obtained using a Broad Ion Beam milling instrument (IM4000, Hitachi, Japan) available at ETH Zürich (ScopeM). Milling was performed using an argon gun accelerated under 6 kV while the sample was wobbled at a middle speed (C3) to avoid heating. Electron microscopy images were acquired after deposition of a 5 nm layer of platinum on the nanovaterite powder and on the polished compact surface (LEO1530, Zeiss, Germany). Transmission electron microscopy images were acquired on FEI Talos F200X.

### Creep tests

The suspension of nanovaterite agglomerates in ethanol was first slip cast in a gypsum mould to remove the excess of ethanol. The powder obtained was dried at 100 °C for 2 h. To perform the compaction experiments, 0.3 g of nanovaterite powder was added into the cavity of a pressing tool of 11 mm diameter (P/O/Weber, Germany). The desired amount of liquid was added directly on top of the powder. A liquid-to-powder (L/P) weight ratio of 0.2 was used. This ratio was typically obtained by mixing 0.060 g of liquid with 0.300 g of powder. The liquid used for most creep tests consisted of a 0.9 wt% aqueous solution of NaCl (EMSURE, Merck). This NaCl concentration is known to increase the CaCO_3_ solubility^36^. Paraffin oil (Sigma-Aldrich) was used in selected experiments to test the effect of the type of liquid on the compaction behaviour. To investigate the creep response of the nanovaterite powder, the pressing tool was closed and placed in a universal testing machine (Instron 8562, Instron) equipped with a 100 kN load cell. A preload of 200 N was applied on the specimen to ensure a common starting point for the compaction tests. The compaction pressure was then applied at a rate of 0.5 mm min^−1^ until the maximum load was reached. This was followed by the application of a constant load that was maintained for time periods ranging from 30 min to 1 h. For each load, a background displacement curve was obtained with an empty pressing tool. This displacement was then subtracted from the actual values measured with the powder to remove the contribution of the tools to the total deformation. To calculate the density of the compacted specimens, the powder masses were measured 24 h after pressing to take into account any loss that may have occurred during the compaction process. The thickness and diameter of the samples were deduced from the displacement of the machine and the diameter of the pressing tool, respectively. Archimedes measurements confirmed that the geometric density measured during the tests were correct within +/−5%.

### Grain size analysis

Grain size measurements were performed using image analysis with the freely available software Fiji on cross-section images of vaterite specimens at different densities. A detailed procedure and examples are given in the Supplementary Information.

### Strain rate modelling

The calculation of the theoretical strain rate of the vaterite samples during compaction was performed using the established pressure solution creep model outlined by Zhang *et al*.^15^ A detailed procedure and Matlab scripts are given in the Supplementary Information.

### Mechanical testing

Samples for mechanical testing were prepared following the same overall protocol used for the creep tests but upscaled for a larger amount of powder (typically 0.5 g). In this case, compaction was carried out using a 13 mm diameter pressing tool in an uniaxial press (200 kN capacity, P/O/Weber, Germany) at various loads for at least 2 h. After pressing, samples were removed and dried for 2 h at room temperature. A typical sample showed a diameter of 13 mm and was 2.2 mm thick, but larger specimens were also obtained (Supplementary Fig. 9). Such disks were cut with a 300 μm wire saw to generate beams of approximately 11 × 2.2 × 1.8 mm^3^ (length × depth × width) and cuboids of approximately 1.7 × 1.7 × 2 mm^3^ for the three-point bending and compression tests, respectively. Samples for bending tests were beveled at the edges and used directly after cutting. All the tests were performed with a Instron 8562 universal testing machine equipped with a 1 kN load cell. A three-point bending set-up with a span of 9.4 mm and a constant loading speed of 1 μm s^−1^ was utilized. The beam deflection was measured using a linear variable differential transducer set-up. Compression experiments were also performed at a constant displacement speed of 1 μm s^−1^. Representative curves for each test are plotted in Supplementary Fig. 10. At least three specimens were tested for each composition. The reported values are averages and standard deviations thereof.

### Data availability

The data that support the conclusions presented in this paper are available from the corresponding author on request.

## Additional information

**How to cite this article:** Bouville, F. & Studart, A. R. Geologically-inspired strong bulk ceramics made with water at room temperature. *Nat. Commun.* **8,** 14655 doi: 10.1038/ncomms14655 (2017).

**Publisher's note**: Springer Nature remains neutral with regard to jurisdictional claims in published maps and institutional affiliations.

## Supplementary Material

Supplementary Information Supplementary Figures, Supplementary Table, Supplementary Methods and Supplementary References

## References

[b1] Aizenberg J., Muller D. A., Grazul J. L. & Hamann D. R. Direct fabrication of large micropatterned single crystals. Science 299, 1205–1208 (2003). 12595685 10.1126/science.1079204

[b2] Yoshimura M. Importance of soft solution processing for advanced inorganic materials. J. Mater. Res. 13, 796–802 (1998).

[b3] Aizenberg J. New nanofabrication strategies: inspired by biomineralization. MRS Bull. 35, 323–330 (2010).

[b4] Kim Y.-Y. . Tuning hardness in calcite by incorporation of amino acids. Nat. Mater. 15, 903–910 (2016). 27135858 10.1038/nmat4631

[b5] Wang T., Cölfen H. & Antonietti M. Nonclassical crystallization: mesocrystals and morphology change of CaCO_3_ crystals in the presence of a polyelectrolyte additive. J. Am. Chem. Soc. 127, 3246–3247 (2005). 15755119 10.1021/ja045331g

[b6] Fratzl P. & Weinkamer R. Nature's hierarchical materials. Prog. Mater. Sci. 52, 1263–1334 (2007).

[b7] Colman S. M. & Dethier D. P. Rates of Chemical Weathering of Rocks & Minerals Academic Press (1986).

[b8] Lim X. How to make the most of carbon dioxide. Nature 526, 628–630 (2015). 26511561 10.1038/526628a

[b9] Guo J. . Cold sintering process of composites: bridging the processing temperature gap of ceramic and polymer materials. Adv. Funct. Mater. 26, 7115–7121 (2016).

[b10] Guo J. . Cold sintering: a paradigm shift for processing and integration of ceramics. Angew. Chem. Int. Ed. Engl. 55, 11457–11461 (2016). 27513705 10.1002/anie.201605443

[b11] Shotton E. & Rees J. E. The compaction properties of sodium chloride in the presence of moisture. J. Pharm. Pharmacol. 18, 160S–167S (1966).

[b12] Gratier J., Dysthe D. K. & Renard F. The role of pressure solution creep in the ductility of the Earth's upper crust. Adv. Geophys. 54, 1–112 (2013).

[b13] Plummer L. N. & Busenberg E. The solubilities of calcite, aragonite and vaterite in CO_2_-H_2_O solutions between 0 and 90 °C, and an evaluation of the aqueous model for the system CaCO_3_-CO_2_-H_2_O. Geochim. Cosmochim. Acta 46, 1011–1040 (1982).

[b14] Rutter E. H. & Elliott D. The kinetics of rock deformation by pressure solution [and discussion]. Philos. Trans. R. Soc. A Math. Phys. Eng. Sci. 283, 203–219 (1976).

[b15] Zhang X., Spiers C. J. & Peach C. J. Compaction creep of wet granular calcite by pressure solution at 28 °C to 150 °C. J. Geophys. Res. 115, B09217 (2010).

[b16] Croizé D., Renard F. & Gratier J. Advances in Geophysics Vol. 54 181–238Elsevier Inc., (2013).

[b17] Renard F., Ortoleva P. & Gratier J. P. Pressure solution in sandstones: influence of clays and dependence on temperature and stress. Tectonophysics 280, 257–266 (1997).

[b18] Rutter E. H. The influence of interstitial water on the rheological behaviour of calcite rocks. Tectonophysics 14, 13–33 (1972).

[b19] de Meer S. & Spiers C. J. Influence of pore-fluid salinity on pressure solution creep in gypsum. Tectonophysics 308, 311–330 (1999).

[b20] Zhang X. & Spiers C. J. Effects of phosphate ions on intergranular pressure solution in calcite: an experimental study. Geochim. Cosmochim. Acta 69, 5681–5691 (2005).

[b21] Wanless H. R. Limestone response to stress: pressure solution and dolomitization. SEPM J. Sediment. Res. 49, 437–462 (1979).

[b22] German R. M. Sintering: from Empirical Observations to Scientific Principles Elsevier (2014).

[b23] Croizé D., Bjørlykke K., Jahren J. & Renard F. Experimental mechanical and chemical compaction of carbonate sand. J. Geophys. Res. 115, B11204 (2010).

[b24] Rutter E. H. Pressure solution in nature, theory and experiment. J. Geol. Soc. London 140, 725–740 (1983).

[b25] Cahn R. W. Nanostructured materials. Nature 348, 389–390 (1990).

[b26] Dargatz B., Gonzalez-Julian J. & Guillon O. Improved compaction of ZnO nano-powder triggered by the presence of acetate and its effect on sintering. Sci. Technol. Adv. Mater. 16, 25008 (2015). 10.1088/1468-6996/16/2/025008 PMC5036464 27877777

[b27] Grossin D. . Biomimetic apatite sintered at very low temperature by spark plasma sintering: physico-chemistry and microstructure aspects. Acta Biomater. 6, 577–585 (2010). 19686872 10.1016/j.actbio.2009.08.021

[b28] Gutmanas E. Y., Rabinkin A. & Roitberg M. Cold sintering under high pressure. Scr. Metall. 13, 11–15 (1979).

[b29] Silva P., Bucea L., Sirivivatnanon V. & Moorehead D. R. Carbonate binders by ‘cold sintering' of calcium carbonate. J. Mater. Sci. 42, 6792–6797 (2007).

[b30] Parakhonskiy B. V., Haase A. & Antolini R. Sub-micrometer vaterite containers: synthesis, substance loading, and release. Angew. Chem. Int. Ed. Engl 51, 1195–1197 (2012). 22375283 10.1002/anie.201104316

[b31] Reed J. S. Principles of Ceramic Processing Wiley (1995).

[b32] Fett T. & Munz D. Ceramics - Mechanical Properties, Failure Behaviour, Materials Selection Springer (1999).

[b33] Lenel F. V. Powder Metallurgy: Principles and Applications Metal Powder Industries Federation (1980).

[b34] Kingery W. D. Densification during sintering in the presence of a liquid phase. I. Theory. J. Appl. Phys. 30, 301–306 (1959).

[b35] Spiers C. J., Meer de S., Niemeijer A. R. & Zhang X. in *Physicochemistry of Water in Geological and Biological Systems* (eds Nakashima, S. .) 129–158Universal Academy Press, Tokyo (2004).

[b36] Zhang X. & Spiers C. J. Compaction of granular calcite by pressure solution at room temperature and effects of pore fluid chemistry. Int. J. Rock Mech. Min. Sci. 42, 950–960 (2005).

[b37] Frey R. G. & Halloran J. W. Compaction behavior of spray-dried alumina. J. Am. Ceram. Soc. 67, 199–203 (2006).

[b38] Wegst U. G. K., Bai H., Saiz E., Tomsia A. P. & Ritchie R. O. Bioinspired structural materials. Nat. Mater. 14, 23–36 (2014). 25344782 10.1038/nmat4089

