# Supplementary material for: Geologically-inspired strong bulk ceramics made with water at room temperature
Source: Nat Commun. 2017 Mar 6;8:14655. doi: 10.1038/ncomms14655 (PMC5343517; doi:10.1038/ncomms14655)
Supplement: Supplementary Information — Supplementary Figures, Supplementary Table, Supplementary Methods and Supplementary References [file ncomms14655-s1.pdf]

Supplementary Figures

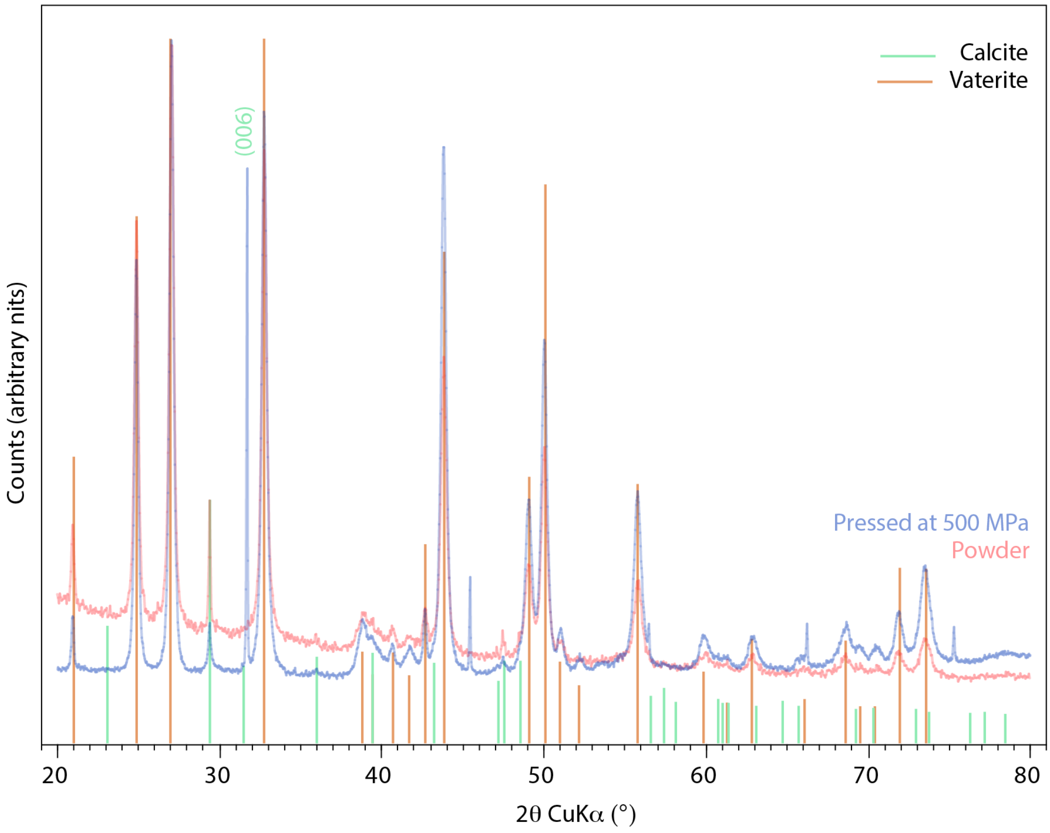

Supplementary Fig. 1 | X-ray diffraction of the synthesized powder and of a cold sintered sample pressed at 500MPa. The powder is a mixture of vaterite and calcite. An average vaterite crystallite size of 37 nm was obtained by fitting the Scherrer equation to the three most intense Bragg peaks below 40°: (110), (112) and (114). The XRD patterns were recorded on an X'Pert Pro powder diffractometer (PANalytical B. V., Netherlands) operated in reflection mode with Cu K α radiation (45 kV, 40 mA). The XRD pattern for the powder matches well the peak relative intensities expected for the vaterite phase. For the pressed samples, an additional peak corresponding to the crystallographic plane of Miller indices (006) of calcite at an angle of 31.43° is observed. The rest of the peaks are similar to the pristine (unpressed) sample. This indicates that the phases ratio in the powder and pressed specimens remain similar within the accuracy of the measurement. The presence of an intense peak corresponding to the (006) plane of calcite indicates a strong texture of those particles (the intensity should be of only 2% according to the JCPDS file). By contrast, vaterite particles do not exhibit any texture. Pressing is known to induce a preferential orientation in powders containing anisotropic (or at least faceted) particles, which explains why we orient the large cubic shaped calcite particles while the almost spherical vaterite ones still present a random crystallographic orientation. Interestingly this texture is also present in natural calcite mineral deformed by pressure in the Earth's crust[1].

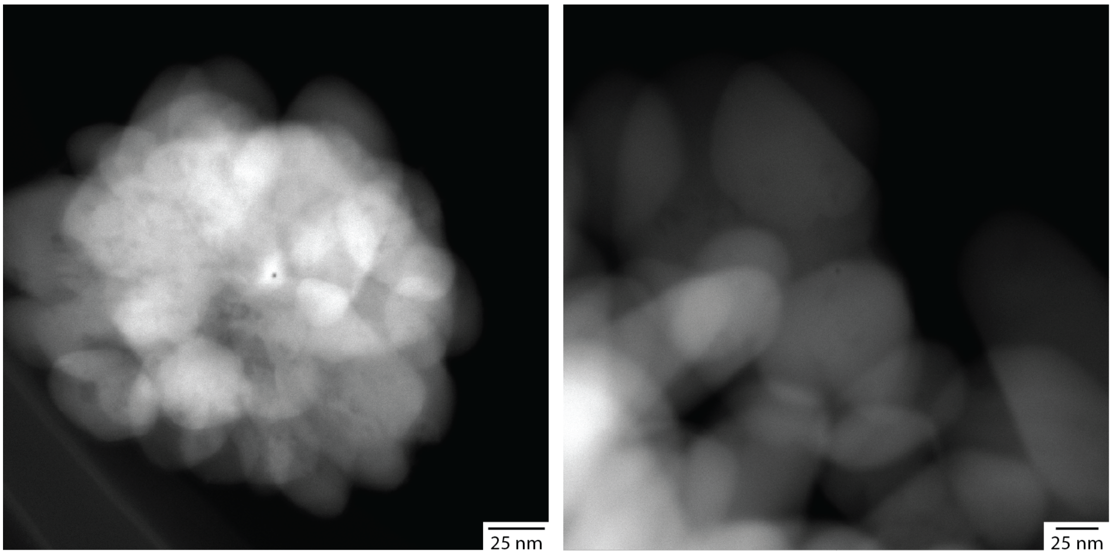

Supplementary Fig. 2 | TEM observations at two magnifications (225k X and 320k X) of the nanovaterite powder.

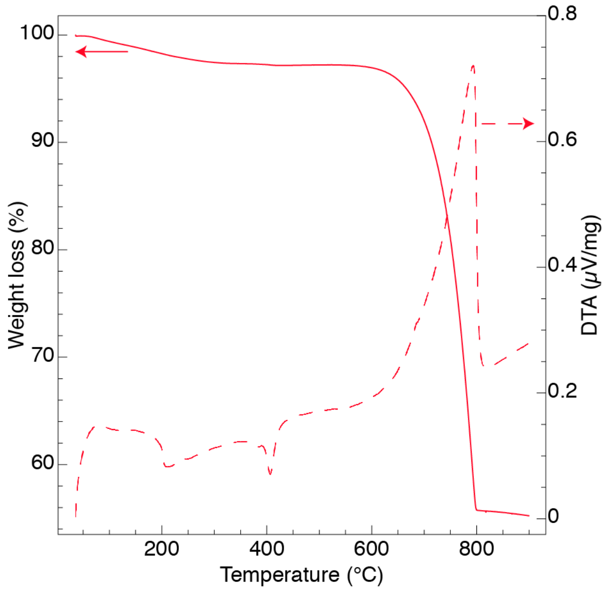

Supplementary Fig. 3 | Thermogravimetric and differential thermal analysis of the nanovaterite powder. The synthesised powder was slip cast to remove the excess of ethanol and dried at room temperature and pressure overnight. A 4 wt% weight loss above 100°C can be observed and is probably due to residual solvent desorption. The second weight loss of 42 wt% is associated with the transformation from the carbonate $CaCO_3$ to the oxide CaO, releasing CO_2. The endothermic peak measured at this temperature supports the occurrence of this reaction. The exothermic peak around 400°C is tentatively associated with the transformation of vaterite into calcite during heating[2]. The test was performed at a heating rate of 5°C/min from 35°C to 900°C under a 80:20 N_2:O_2 mixture with a TGA-DSC device (STA 449 C, Netzch).

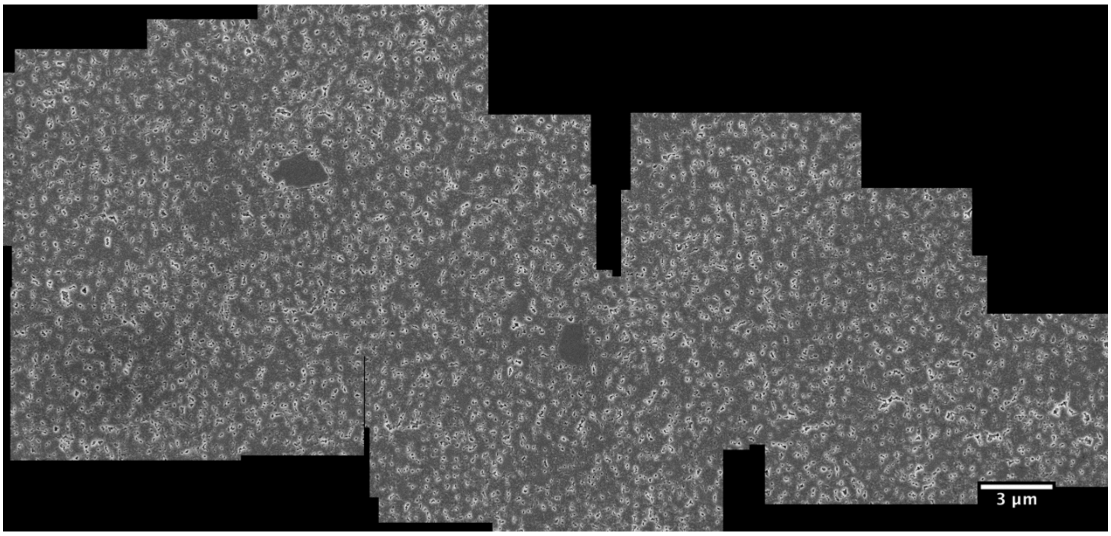

Supplementary Fig. 4 | Example of 30 SEM images stitched together for grain size analysis of a sample pressed at 500 MPa.

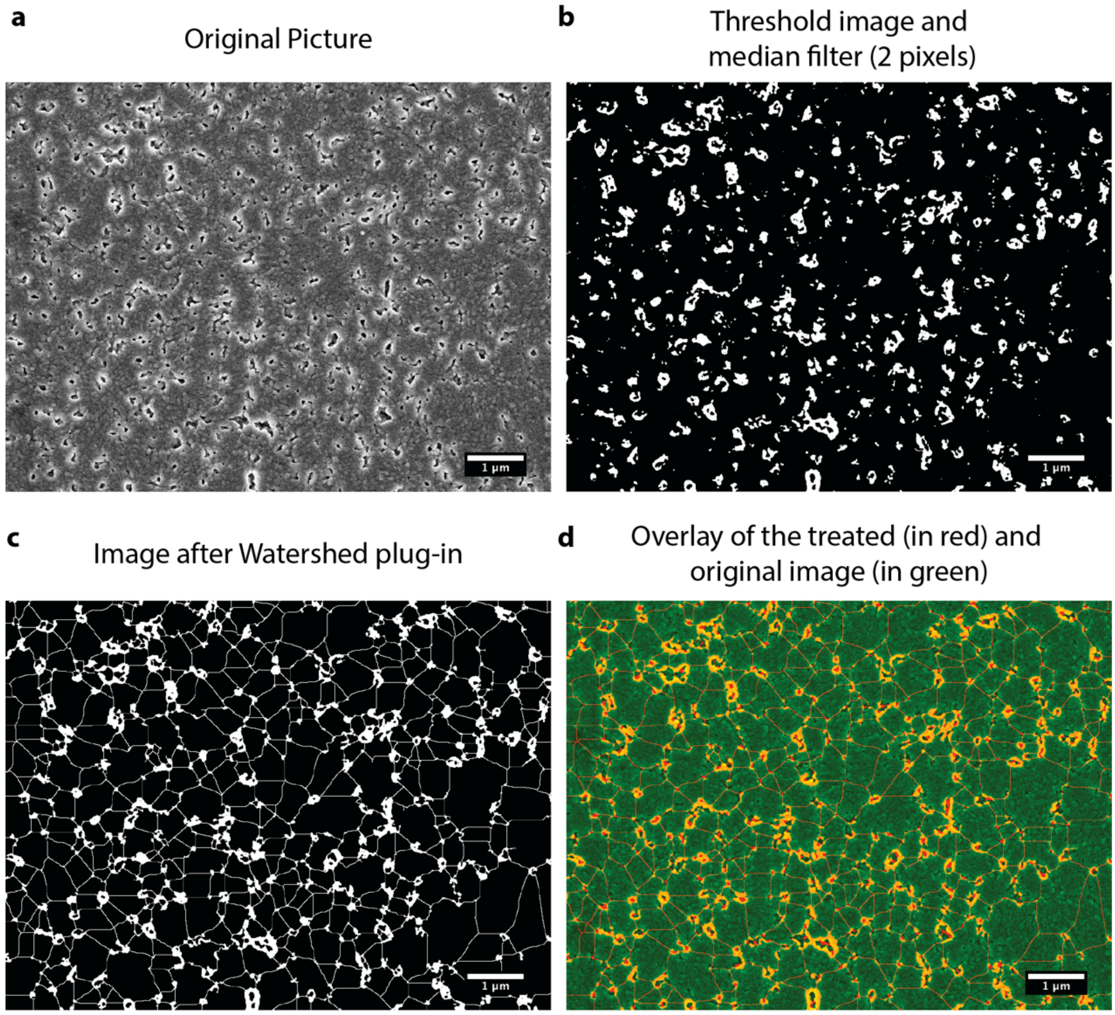

Supplementary Fig 5 | Example of grain size measurement procedure on a SEM picture of a sample pressed at 500 MPa.

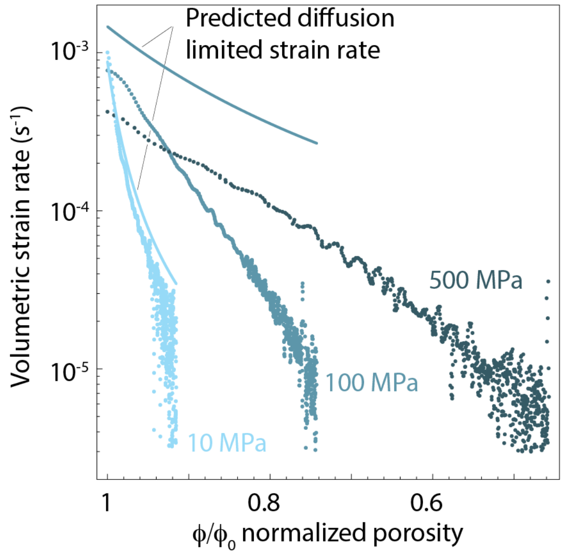

Supplementary Fig. 6 | Experimental strain rates obtained for three applied stresses along with the model predictions for lower stresses of 10 MPa and 100 MPa. The model accurately predicts the strain rate at an applied stress of 10 MPa but highly overestimates the rate if the stress is increased to 100 MPa.

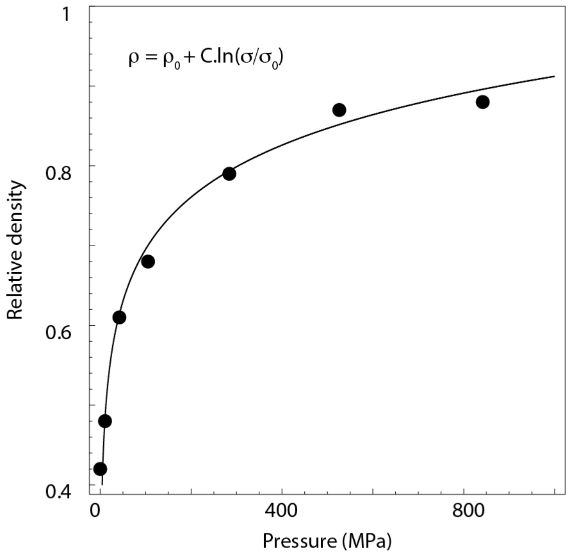

Supplementary Fig 7 | Logarithmic dependence of the relative density of vaterite compacts on the applied stress after a compaction time of at least 1800s. The values of ρ_0, σ_0 and C are 0.48, 10.5 MPa and 0.094 respectively**.**

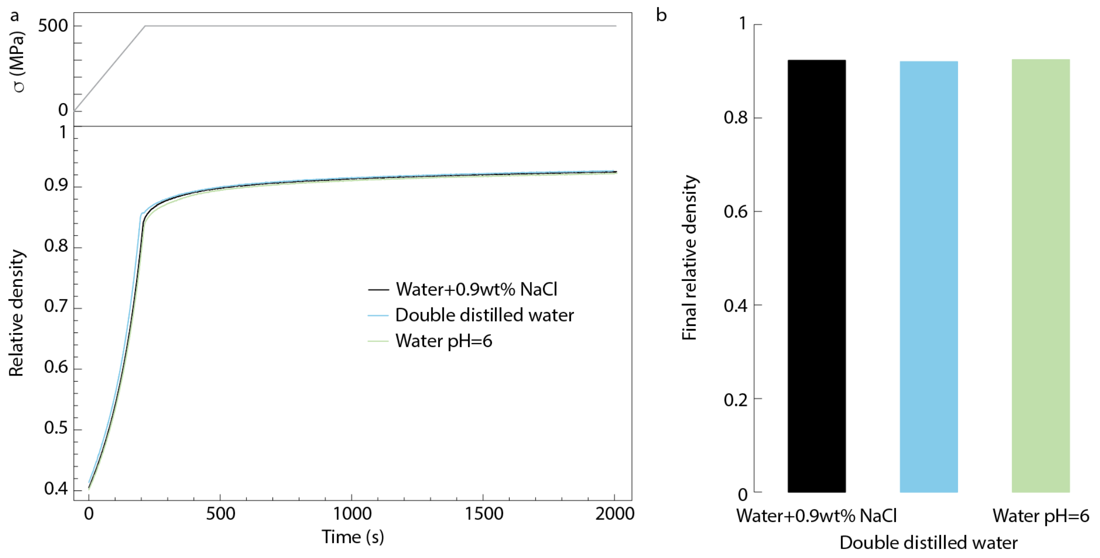

Supplementary Fig. 8 | Effect of the medium composition on the densification kinetics at an applied pressure of 500 MPa. a. Evolution of the relative density as a function of time. The applied stress profile is plotted in the top part. b. Final relative densities obtained with different liquid media.

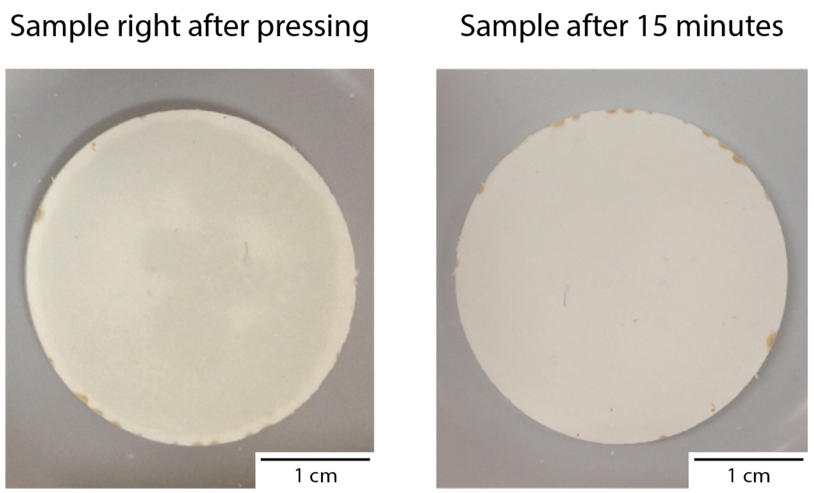

Supplementary Fig. 9 | Pictures of a large sample with a diameter of 30 mm just after pressing at 280 MPa and 15 minutes later. The color change indicates that water is still present in the sample even at high pressures. After removal of the specimen from the mold, water evaporates in a few minutes.

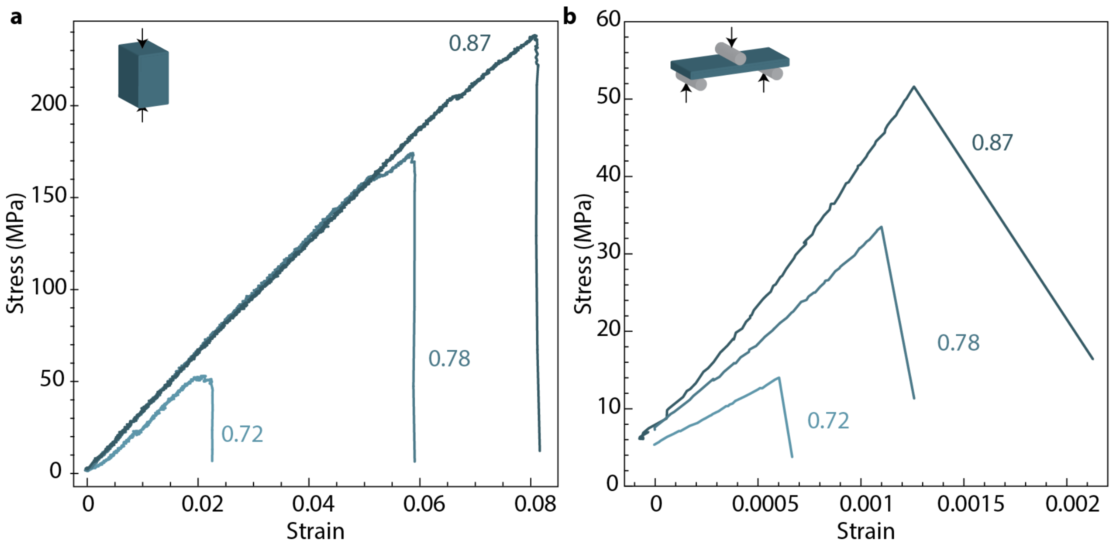

Supplementary Fig. 10 | Typical stress-strain curves obtained for cold sintered samples under compression (a) and three-point bending (b). The average relative densities of the samples are written alongside the curves.

Supplementary Table

Supplementary Table 1 | Parameters used for the strain rate calculation

Parameter, description	Value (units)	Reference
D, average grain boundary diffusivity	$1x10^{-14}$ (m^2/s)	[8]
C, calcite solubility at 25°C	$5.8x10^{-5}$ (kg/m^3)	[6]
S, size of the liquid film between grains	$1x10^{-9}$ (m)	[9]
σ_e, applied stress	variable (Pa)	
Ω, molar volume	$3.692x10^{-5}$	
R, universal gas constant	8.314 (J / Mol / K)	
T, temperature	298 (K)	
d, average grain size D_{50} from Fig. 3d	$600x10^{-9}$ (m)	

Supplementary Methods

Grain size measurements by image analysis

The grain size of the cold sintered samples was measured with the freely available software Fiji[3]. Several SEM images were stitched together with a dedicated plug-in[4] to obtain a larger number of grains (Supplementary Fig. 4) while keeping a resolution high enough to distinguished the pores and grain boundaries. A simple threshold was used to separate the pores from particles (Supplementary Fig. 5b), followed by a 2 pixels median filtering procedure to remove noise in the image. The plug-in Watershed[5] was then used to join the pores together and thus retrace the grain boundaries (Supplementary Fig. 5c). The resulting grain boundaries and the original image were overlaid to check the accuracy of the method (Supplementary Fig. 5d). The plug-in “Analyse Particles” was finally used to obtain the grain size distribution plotted in Fig. 3d of the main manuscript.

Description of the model for strain rate estimation and copy of the Matlab scripts

The model used to estimate the strain rate during powder compaction is based on the analysis of the chemical potential equilibrium between a stressed grain and a pressurized liquid, as recently described by Zhang *et al.*[6] [7]. From the three different processes of dissolution, diffusion and precipitation occurring during pressure solution creep (Fig. 1a in the main manuscript), the diffusion step is assumed to be the kinetically limiting one[6]. This model has been mostly used for calcite particles. Here, we applied it to vaterite particles utilizing parameters available in the literature. The following equation (S1) was used to calculate the volumetric strain rate $\dot{\varepsilon_d}$ associated with grain boundary diffusion:

$$\dot{\varepsilon_d} = DCS\frac{\exp\left(\frac{B\sigma_e\Omega}{RT}-1\right)}{d^3}f_d(\phi_0, e_v) \qquad \text{(S1)}$$

where ϕ_0 is the initial porosity and e_v is the volumetric strain. The other variables are described in Supplementary Table 1.

The functions $f_d(\phi_0, e_v)$ and $B(\phi_0, e_v)$ in equation S1 account for the change in grain contact morphology during shrinkage of the compact.

$f_d(\phi_0, e_v)$ represents the evolution of the grain boundary fraction during compaction, assuming the grains to be spheres packed in a simple cubic arrangement. This leads to the following function:

$$f_d(\phi_0, e_v) = \frac{144\,\pi\,d^2}{A_c(1-e_v)^{1/3}} \quad \text{(S2)}$$

where A_c is the contact area per grain at a strain e_v, expressed as:

$$A_c = 6\,\pi\,\left(\frac{d^2}{4} - x^2\right), \quad \text{(S3)}$$

and x is the distance between the grain center and the grain boundary, expressed as:

$$x = \frac{d}{2}(1 - e_v)^{1/3}. \quad \text{(S4)}$$

$B(\phi_0, e_v)$ represents the stress intensification factor at the grain contact and is defined as:

$$B(\phi_0, e_v) = \frac{6\,d^2(1-e_v)^{2/3}}{A_C}. \quad \text{(S5)}$$

Scripts for calculating strain rate under compaction

The calculation of the strain rate during compaction utilizes two files. In one file the user provides the different model parameters and experimental strain data as input data and a function is called to calculate the theoretical strain rate at each strain level. The second file is the function itself. The scripts shown below can be copied and pasted directly in Matlab and are ready to use.

Script:

```
clear all

%% Allocate imported array to column variable names --> replace with your data here
e = data(:,1); % Vector containing the strain measured and corrected
epexp = data(:,2); Vector containing experimental strain rate to compare with
P = data(1,3); % initial relative density
Se= data(1,4); %applied stress

d=600e-9;           %Particles diameter m
phi0=1-P;           %porosity
Dbb=1e-14;          %Grain boundary diffusivity average m2/s [Nakashima,1995]
S=1e-9;             %size of liquid film m [Rutter,1976]
T=25;               %temperature of the system ?C
lK=-171.9065-0.077993*(T+273.15) + 2839.319/(T+273.15) + 71.595*log10(T+273.15);
C=(10^(lK))^(1/2);  %Solubility of calcite from solubility product and temperature
rho=2540;           %density of vaterite kg/m3
om=3.692e-5;        %molar volume
```

```
for i=1:size(e,1)
ev(i)=e(i,1);
[epdiff(i),phi(i)] = EpsDiss(d,ev(i),phi0,Se,Db0,H,S,C,T,rho,om,Dbb);
end
loglog(ev,epexp,'o',ev,epdiff,'--')
```

Function EpsDiss:

```
function [epdiff,phi] = EpsDiss(d,ev,phi0,Se,Db0,H,S,C,T,rho,om,Dbb)

% Created by F Bouville, Complex Materials, ETH Zurich, 2015
% Calculation of the volumetric strain rate in pressure solution creep,
% according to Zhang et al. 2010,  doi: 10.1029/2008JB005853
% Assumptions : grains are spheres of diameter d and present a simple cubic
% packing, T in Celsius, ev is the axial strain (equal to volume strain in a
% compaction experiment),related to the density phi/phi0 =(1 - ev/phi0)/(1 - ev)

x=d/2*(1-ev)^(1/3); %x is the distance grain center to grain contact
Ac=6*pi*(d^2/4-x^2); %contact area per grain at ev

R=8.314; %Perfect gas constant, J/K/mol
phi=phi0*(1-ev/phi0)/(1-ev); %porosity

B=6*d^2*(1-ev)^(2/3)/Ac; %Stress intensification factor at grain contact at ev+

fd=144*pi*d^2/(Ac*(1-ev)^1/3); %porosity function for grain boundary diffusion

epdiff=fd*Dbb*C*S/(d^3)*(exp(B*Se*om/(R*(T+273.15)))-1);

end
```

Consideration of sample size effects on the mechanical properties

Since the vaterite samples prepared in this work are considerably smaller than concrete specimens typically used for mechanical testing, Weibull analysis was used to estimate the strength of concrete if it were to show similar dimensions to the vaterite compacts. Based on Weibull theory, the strength of a brittle material (σ) scales with its characteristic dimension D, as follows[10]:

$\sigma \propto D^{-3/m}$, where m is the Weibull modulus.

Taking into account a Weibull modulus of 12 for concrete[11], we estimate that a concrete sample with dimension comparable to that of the vaterite compact (2 mm) should be

about two-fold stronger than a concrete specimen with typical macroscopic dimensions (30 mm). This increase in strength does not affect the order-of-magnitude comparison of mechanical properties shown in the Ashby diagram (Fig. 4b, main text).

Supplementary References

1. Wenk, H. R., Takeshita, T., Bechler, E., Erskine, B. G. & Matthies, S. Pure shear and simple shear calcite textures. Comparison of experimental, theoretical and natural data. *J. Struct. Geol.* **9,** 731–745 (1987).
2. Perić, J., Vučak, M., Krstulović, R., Brečević, L. & Kralj, D. Phase transformation of calcium carbonate polymorphs. *Thermochim. Acta* **277,** 175–186 (1996).
3. Schindelin, J. *et al.* Fiji: an open-source platform for biological-image analysis. *Nat. Methods* **9,** 676–82 (2012).
4. Preibisch, S., Saalfeld, S. & Tomancak, P. Globally optimal stitching of tiled 3D microscopic image acquisitions. *Bioinformatics* **25,** 1463–1465 (2009).
5. Vincent, L. & Soille, P. Watersheds in digital spaces: an efficient algorithm based on immersion simulations. *IEEE Trans. Pattern Anal. Mach. Intell.* **13,** 583–598 (1991).
6. Zhang, X., Spiers, C. J. & Peach, C. J. Compaction creep of wet granular calcite by pressure solution at 28°C to 150°C. *J. Geophys. Res.* **115,** B09217 (2010).
7. Renard, F., Ortoleva, P. & Gratier, J. P. Pressure solution in sandstones: influence of clays and dependence on temperature and stress. *Tectonophysics* **280,** 257–266 (1997).
8. Nakashima, S. Diffusivity of ions in pore water as a quantitative basis for rock deformation rate estimates. *Tectonophysics* **245,** 185–203 (1995).
9. Rutter, E. H. & Elliott, D. The Kinetics of Rock Deformation by Pressure Solution [and Discussion]. *Philos. Trans. R. Soc. A Math. Phys. Eng. Sci.* **283,** 203–219 (1976).
10. Van Mier, J. G. . & Van Vliet, M. R. . Influence of microstructure of concrete on size/scale effects in tensile fracture. *Eng. Fract. Mech.* **70,** 2281–2306 (2003).
11. Zech, B. & Wittmann, F. H. Part II Probabilistic approach to describe the behaviour of materials. *Nucl. Eng. Des.* **48,** 575–584 (1978).
